# Supplementary material for: The causal relationship between the human gut microbiota and pyogenic arthritis: a Mendelian randomization study
Source: Front Cell Infect Microbiol. 2024 Nov 26;14:1452480. doi: 10.3389/fcimb.2024.1452480 (PMC11629706; doi:10.3389/fcimb.2024.1452480)
Supplement: Supplementary file 1 [file DataSheet1.zip › mendelian test/ebi-a-GCST90027809.csv_forest.pdf]

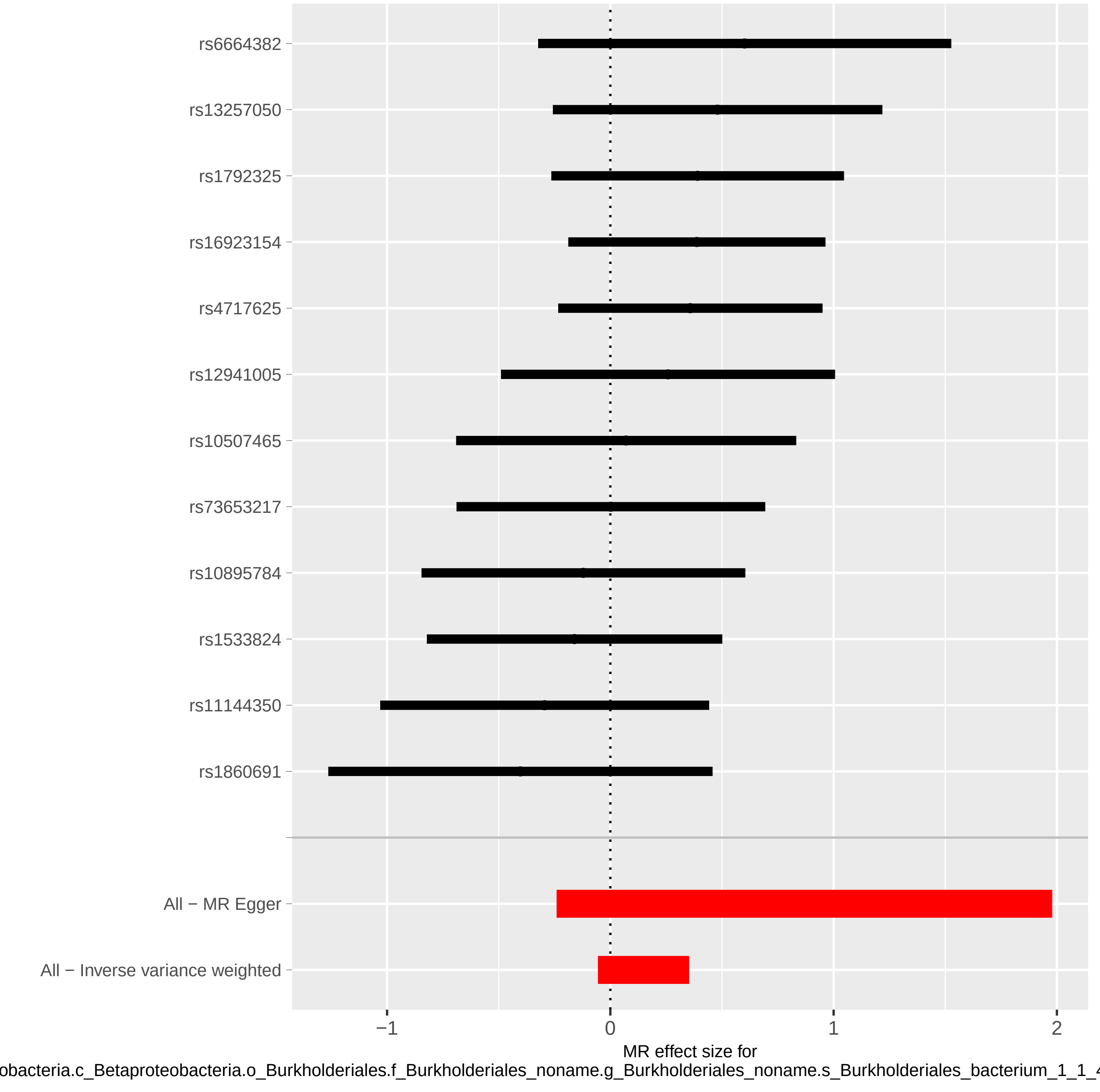
rs6664382
rs13257050
rs1792325
rs16923154
rs4717625
rs12941005
rs10507465
rs73653217
rs10895784
rs1533824
rs11144350
rs1860691
All − MR Egger
All − Inverse variance weighted
−1
0
1
2
MR effect size for
obacteria.c_Betaproteobacteria.o_Burkholderiales.f_Burkholderiales_noname.g_Burkholderiales_noname.s_Burkholderiales_bacterium_1_1_4
